# A positive side effect of wearing face coverings for socially anxious females: Findings from a speech task

**DOI:** 10.1016/j.heliyon.2023.e23733

**Published:** 2023-12-15

**Authors:** Carina Tiewald, Arved Seibel, Anne Schienle

**Affiliations:** Department of Clinical Psychology, University of Graz, Graz, Austria

**Keywords:** Social anxiety, Face masks, Behavioral assessment, Physiological assessment

## Abstract

**Background:**

Wearing face masks has become more common due to the COVID-19 pandemic. Until now, it has not been investigated whether socially anxious individuals can reduce their acute anxiety symptoms by wearing face masks during a speech task.

**Method:**

Fifty-nine socially anxious females were asked to prepare and give an oral presentation. Participants were randomly allocated either to a group that was asked to wear a face mask during the task, or to a group that was not asked to wear a face mask during the task. Dependent variables included physiological parameters (systolic/diastolic blood pressure, heart rate) and self-reports (valence and arousal at baseline, as well as directly before and after the presentation).

**Results:**

The analyses showed that the group without face masks had a higher systolic blood pressure than the group with face masks, directly before as well as after the presentation. The two groups did not differ in the other measures.

**Conclusion:**

Wearing face masks has a small stress-reducing effect on socially anxious females.

## 1 Introduction

Wearing protective face coverings in public spaces has played a vital role in mitigating the spread of the coronavirus. Aside from this advantageous effect, however, face masks can also bring about unintended adverse effects, such as in the realm of social communication. The concealment of the nose/mouth region impairs speech intelligibility [1,2] and the ability to make accurate inferences about facial expressions or the gaze of others [[3], [4], [5], [6]]; but also see Refs. [7,8]. This may lead to uncertainty and even distress in the observer, which may be further exacerbated in the presence of mental health problems. For example, a survey by Carney et al. [9] demonstrated that elevated anxiety symptoms were associated with more physical and emotional discomfort related to mask-wearing.

On the other hand, there is also some evidence suggesting possible psychological benefits of wearing face masks. For instance, regularly wearing a mask (vs. no mask) has been associated with lower levels of reported anxiety, depression, and somatic symptoms [10,11]. Further, the wearing of face masks may also bestow immediate positive effects: In one experiment, participants with or without wearing a mask viewed negative facial expressions presented on a computer screen [12]. They were made to believe they were being watched by an audience via webcam. Participants with a mask (who were able to hide their feelings) reported experiencing fewer negative emotions in this social-evaluative setting.

This possibility of hiding negative emotions may be particularly compelling for people with social anxiety. The main symptom of social anxiety disorder (SAD) is an intense, persistent fear of being watched and negatively judged by others [13]. A person with SAD experiences psychological and somatic symptoms of anxiety in social situations, such as speaking in public, meeting new people, or answering questions in class or during a job interview. People with social anxiety feel self-conscious, criticize their social skills, and worry that others may see them as incompetent. Moreover, they worry that others will notice their physical anxiety symptoms, including blushing, sweating, and anxious facial expressions.

Individuals with elevated social anxiety often engage in a variety of internal as well as external safety behaviors that aim at reducing anxiety in feared social situations (e.g., excessive memorization before a speech, and attending a party but only speaking to familiar people). These safety behaviors also include attempts at hiding physical anxiety symptoms via make-up or clothes to conceal blushing and sweating. Mask-wearing may fulfill a similar function [14]. Due to the partial concealment of the face with a mask, anxiety symptoms cannot be detected as easily by others, a factor that might be stress-reducing for people with social anxiety.

The present experiment investigated whether wearing a face mask during a speaking task would reduce stress symptoms in individuals high in social anxiety. In recruiting anxious participants for the study, a cover story was created: Participants were invited to give a brief oral presentation on the negative consequences of COVID-related restrictions they experienced in their daily lives. The study was labeled ‘Vent Your Frustrations’. Before and after the oral presentation, participants' ratings for their affective state (valence, arousal) as well as their performance were assessed.

Moreover, physiological measures (blood pressure/pulse) were recorded. These parameters are frequently used markers of the somatic stress response displayed during speaking tasks containing a social-evaluative component [[15], [16], [17], [18]]. It was hypothesized that mask-wearing would be associated with less reported discomfort as well as decreased blood pressure shortly before and after the speech task.

## 2 Method

### 2.1 Participants

The sample consisted of 59 female students (mean age: M = 21.80 years, SD = 2.62), who reported elevated social anxiety (see Table 1). The sample was restricted to females to reduce sex-related variance. The prevalence of social anxiety and SAD is higher in the female population [19,20]. Moreover, stress responses differ between males and females (e.g. Ref. [21]). Regarding our sample size, we were guided by studies that used a similar design [16,22].

**Table 1:** Descriptive statistics (means, standard deviation) and group comparisons.

Measure	Group without masks (n – 33) M (SD)	Group with masks (n – 26) M (SD)	t(p)
			
Age	21.36 (2.09)	22.35 (3.12)	−1.38 (.175)
Body mass index	21.56 (2.64)	21.13 (2.98)	0.59 (.560)
*Questionnaires*			
BSI-18	13.30 (8.06)	14.00 (7.84)	−0.33 (.740)
BSI_SOM	2.42 (2.09)	3.04 (3.47)	−0.84 (.403)
BSI_DEP	5.42 (4.29)	6.00 (3.57)	−0.55 (.584)
BSI_ANX	5.45 (3.59)	4.96 (3.56)	0.53 (.601)
SIAS	32.76 (11.56)	32.00 (12.70)	0.24 (.812)
BFNE_R	41.15 (10.12)	40.62 (9.83)	0.21 (.839)
*Baseline* SBP (mmHg)	121.88 (12.86)	118.12 (10.42)	1.21 (.231)
DBP (mmHg)	77.61 (9.50)	73.69 (8.53)	1.64 (.106)
HR (bpm)	79.91 (15.01)	78.62 (11.56)	0.36 (.718)
Arousal	3.61 (1.60)	2.73 (1.56)	2.11 (.039)
Valence	7.12 (.99)	6.81 (1.20)	1.10 (.277)

Footnote: BSI-18: Brief Symptom Inventory-18; BSI_SOM: BSI-Subscale Somatization; BSI_DEP: BSI-Subscale Depression; BSI_ANX: BSI-Subscale Anxiety; SIAS: Social Interaction Anxiety Scale; BFNE_R: Brief Fear of Negative Evaluation – Revised; SBP: Systolic blood pressure; DBP: Diastolic blood pressure; HR: Heart rate.

The participants completed the Social Interaction Anxiety Scale (SIAS; German version [23]) and the Brief Fear of Negative Evaluation Scale – Revised (BFNE_R; German version [24]). The SIAS measures distress when meeting and talking with others. The scale contains 20 items (e.g., ‘I worry about not knowing what to say in social situations’) which are rated on a 5-point scale (0: ‘not at all characteristic of me’; 4: ‘extremely characteristic of me’). A sum score is computed with higher values indicating greater social anxiety. Cronbach's alpha in the present sample was .89. The BFNE_R comprises 12 items (e.g., ‘I worry about what kind of impression I make on people’), that are rated on 5-point Likert scales ranging from 1 (‘not at all characteristic of me’) to 5 (‘extremely characteristic of me’). Cronbach's alpha in the present sample was .93.

Exclusion criteria were less than 22 points on the SIAS and less than 38 points on the BFNE_R, reported somatic diseases (e.g., heart diseases), intake of medication that affects cardiovascular reactivity, high blood pressure (≥140/90 mmHg), obesity (BMI >29), pregnancy and reported mental disorders (except for social anxiety disorder). Self-reported psychological symptoms (somatization, depression, anxiety) were assessed via the Brief Symptom Inventory-18 (BSI-18; German version [25]). Cronbach's alpha of the subscales ranged between 0.69 and 0.79. The T-scores for the subscales were all below the clinical cut-off.

The participants were randomly allocated to one of two groups: oral presentation with face covering (n = 26) or without face covering (n = 33). The groups did not differ in mean age, body mass index, questionnaire scores, and baseline measures, excluding arousal ratings (Table 1).

### 2.2 Procedure

The study complied with all relevant ethical guidelines and regulations involving human participants and was approved by the ethics committee of the University of Graz (Austria; GZ 39/99/63 ex 2020/21). All participants provided written informed consent before participating. Individuals were recruited via postings at the university and social media. The study was conducted during times with COVID restrictions requiring the wearing of a face mask.

The participants answered an online survey that included the BFNE_R, SIAS, BSI-18, and asked for sociodemographic data. All eligible participants were given separate appointments for the experiment (‘Vent your frustration’), which in each case took place in the same university lecture hall.

After a baseline measurement, during which all participants wore face masks, the experimenter left the hall. At this point, one group was instructed to take off their mask. Subsequently, the participants were instructed to prepare an oral presentation (lasting 5 min) in which they were supposed to argue in detail about what was bothering them about the COVID-19 restrictions in their daily lives. For the preparation (3 min), the participants were provided with a piece of paper and a pen to take notes. The participants were not allowed to use these notes during the presentation (the procedure is equivalent to the component of a well-validated social stress test, the Trier Social Stress Test by Kirschbaum et al. [15]).

The presentation was given via a web conference system including a video camera, allowing participants to see themselves (but not the audience), on the computer screen during the experiment. All of the participants were alone in the room when delivering the presentation.

The participants were told that the experimenter would evaluate their performance in terms of the quality of their arguments and presentation style.

As part of the study, self-reports for the affective state (arousal, valence, on 9-point scales reaching from ‘I feel not at all aroused’ to ‘very aroused’ and ‘I feel very bad’ to ‘very good’), systolic/diastolic blood pressure, and pulse (boso medicus uno upper arm blood pressure system; Bosch + Sohn GmbH und Co. KG) were measured three times during the experiment: at baseline (before speech preparation), directly before the presentation, and after the presentation. Furthermore, the participants evaluated their expected performance (plausibility of arguments) on the task (‘*How plausible will you be able to present your arguments?*‘*;* ‘*How plausible will others find your arguments?*’ ‘1–7; 7 = ‘very plausible’) and after the oral presentation (‘*How plausibly have you been able to present your arguments?*‘*;* ‘*How plausible have others found your arguments?*’ 1–7; 7 = ‘very plausible’). At the end of the study, all participants were informed about the goal of the study.

### 2.3 Statistical analysis

The two groups differed in a baseline measure (arousal; Table 1). Therefore, analyses of covariance (ANCOVAs) were conducted to investigate the effect of GROUP (with/without mask) and TIME (pre/post speech) on the dependent variables. In addition, participants' confidence in their ability to produce plausible arguments and participants’ confidence that their arguments would be perceived as such were included as dependent variables in 2 × 2 mixed ANOVAs, each with the factors GROUP and TIME.

Means and standard deviations for all dependent measures over three time periods (baseline, pre/post speech) are provided in the Supplementary Table S1. A validation check concerning task-related stress induction indicated that participants' positive valence rating was significantly higher at baseline compared to the pre-speech period (F (1, 57) = 26.05, p < .001, partial eta squared = 0.314). Additionally, participants’ reported arousal as well as their heart rate were lower at baseline than at pre-speech (arousal: F (1,57) = 74.07. p < .001, partial eta squared = 0.565; heart rate: F (1,57) = 6.01, p = .017, partial eta squared = 0.095).

## 3 Results

**Fig. 1:**
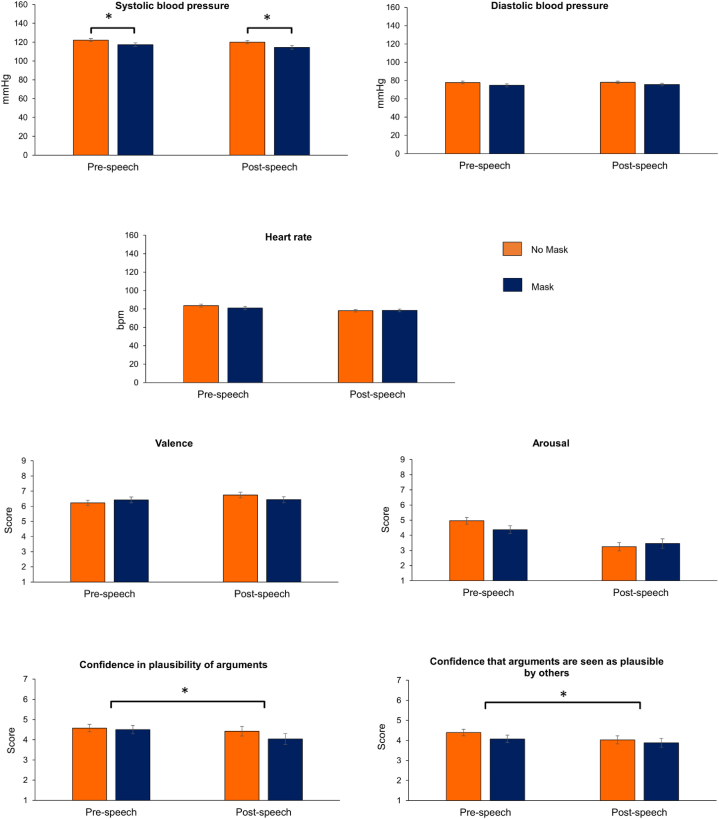
Systolic/diastolic blood pressure, heart rate, valence, arousal, and confidence ratings in the groups with and without mask, before and after the oral presentation F*ootnote*: Means for all measures besides the confidence ratings are adjusted for baseline values. Error bars indicate the standard error. Asterisks indicate a statistically significant difference (p < .050).

The ANCOVA for systolic blood pressure revealed a significant main effect GROUP (F (1,56) = 5.71; p = .020, partial eta squared = 0.092). Before and after the speech, the group without masks had a higher systolic blood pressure than the group with masks (see Fig. 1).

The main effect TIME (F (1,56) = 0.30, p = .585, partial eta squared = 0.005), as well as the interaction effect GROUP x TIME (F (1,56) = 0.09, p = .767, partial eta squared = 0.002) were non-significant. For the other physiological measures and valence/arousal ratings, the ANCOVAs did not detect any statistically significant effects (each p > .05; see Supplementary Table 2).

The ANOVAs for the performance ratings showed that, shortly before their speech, participants were more confident in their ability to produce plausible arguments (F (1,57) = 4.17, p = .046, partial eta squared = 0.068) and have them evaluated as such (F (1,57) = 5.16, p = .027, partial eta squared = 0.083) compared to shortly after their speech. This was the case, independent of the group (neither the main effect GROUP nor the interaction was significant; each p > .050; Supplementary Table S3).

## 4 Discussion

This study investigated if socially anxious females experience less discomfort shortly before and after an oral presentation when wearing a face mask. The masks reduced somatic arousal as indicated by systolic blood pressure. However, subjective ratings (valence, arousal) were not affected by the experimental manipulation.

The Challenge and Threat Theory [26] proposes that people show different cardiovascular reaction patterns in situations that are appraised as challenges (characterized by increased cardiac output and decreased vasoconstriction) vs. ones that are appraised as threats (characterized by reduced cardiac performance and increased vasoconstriction). Although blood pressure is not an optimal indicator for threat appraisal [27], the observed group difference might indicate that participants without face masks felt more exposed to the rater's critical judgment, thus reacting with a more pronounced threat pattern. Based on the model [26], diastolic blood pressure as well as subjective ratings of discomfort should also increase during threat. The incongruence between cardiovascular parameters and affective ratings during social stress tests however has also been reported in previous studies focusing on social anxiety [16,18].

A complementary interpretation of our results can be drawn from Obrist's theory of Active and Passive Coping [28] in combination with the energization model (for a summary [29]). This theory states that stressful situations over which individuals can exert influence produce different cardiovascular reaction patterns than those that have to be endured passively [28]. Active coping is accompanied by an increase in heart rate and/or systolic blood pressure. Furthermore, according to Wright [29], invested effort in a task will increase with perceived task difficulty as long as the achievement of the goal seems attainable and worthwhile. Several studies provided empirical evidence supporting these predictions [30,31]. In the present study, the group without face masks may have exerted more effort than the group with masks as indicated by a higher level of systolic blood pressure. Without the face mask, the task might have been perceived as more demanding resulting in the mobilization of additional resources.

This could have consequences for the development of social anxiety disorder (SAD). If active coping is inhibited when confronted with anxiety-inducing stimuli, habituation will be imapired. Combined with the added uncertainty caused by the masking of facial expressions and gestures of the counterpart, this could contribute to the reinforcement of anxiety symptoms in the long run and ultimately lead to the manifestation of SAD. On the other hand, the immediate stress-reducing effect of the mask could help individuals to engage in social (evaluation) situations more easily and create positive learning experiences.

However, current studies on the impact of the COVID-19 pandemic on the prevalence and incidence of SAD provide heterogeneous results. Besides many other factors, age and individual coping strategies seem to be particularly relevant in this context [32].

The task performance was judged more negatively after the speech independent of the group assignment. This might be related to increased negative rumination after the social stressor [33].

As for the limitations of our study, it is noteworthy that the participants were alone in the lecture hall during the speech. This might have reduced the stress level as indicated by relatively high valence ratings across all measurement periods. However, the participants were able to see themselves on the screen during the experiment and were in auditory communication with the experimenter. As a result, they were constantly aware of being observed and evaluated. The participants displayed increased heart rates and reported more arousal, and more negative valence in the pre-speech period compared to baseline. Thus, the anticipation of the impending speaking task-induced stress in the participants. Furthermore, the participants were not diagnosed with SAD but were characterized by elevated scores on self-report measures. More pronounced effects should be present in clinical groups. Finally, our results cannot be generalized to males, other types of face coverings (e.g., scarfs), and social tasks.

Finally, it is unlikely that the observed group differences resulted from direct effects of the mask on respiratory function, which in turn could change cardiovascular parameters. Previous studies have reported an increase in systolic blood pressure while wearing a face mask, rather than the reduction as observed in the current sample [34].

## 5 Conclusion

To our knowledge, this was the first psychophysiological study to examine the immediate effects of wearing a face mask in a group of socially anxious individuals. The experiment revealed small benefits of wearing a face mask during a speaking task. Further studies are needed to investigate the potential long-term effects of this safety behavior on social anxiety.

### Ethics statement

The study complied with all relevant ethical guidelines and regulations involving human participants and was approved by the ethics committee of the University of Graz (Austria; GZ 39/99/63 ex 2020/21). All participants provided written informed consent before participating.

## Data availability statement

Data associated with this study have not been deposited into a publicly available repository, since the study participants did not give their permission to make their data publicly available.

Data are available from the corresponding author upon request (email).

## CRediT authorship contribution statement

**Carina Tiewald:** Conceptualization. **Arved Seibel:** Writing – original draft, Formal analysis. **Anne Schienle:** Writing – original draft, Supervision.

## Declaration of competing interest

The authors declare that they have no known competing financial interests or personal relationships that could have appeared to influence the work reported in this paper.
