# Supplementary material for: A positive side effect of wearing face coverings for socially anxious females: Findings from a speech task
Source: Heliyon. 2023 Dec 15;10(1):e23733. doi: 10.1016/j.heliyon.2023.e23733 (PMC10767500; doi:10.1016/j.heliyon.2023.e23733)
Supplement: Multimedia component 1 [file mmc1.docx]

**Supplementary Material**

**Table S1** Means and standard deviations of the dependent variables for the two experimental groups over three time periods

| \| Dependent variable \|  \| Group without masks M (SD) \| \| \|  \| Group with masks M (SD) \| \| \| \| --- \| --- \| --- \| --- \| --- \| --- \| --- \| --- \| --- \| \|  \|  \|  \| Period \|  \|  \|  \| Period \|  \| \|  \|  \| Baseline \| Pre-speech \| Post-speech \|  \| Baseline \| Pre-speech \| Post-speech \| \| SBP (mmHg) \|  \| 121.88 (12.86) \| 123.48 (14.22) \| 121.09 (12.11) \|  \| 118.12 (10.42) \| 115.81 (10.47) \| 113.00 (12.46) \| \| DBP (mmHg) \|  \| 77.61 (9.50) \| 78.79 (10.34) \| 79.06 (8.48) \|  \| 73.69 (8.53) \| 73.42 (8.87) \| 74.19 (9.16) \| \| HR (bpm) \|  \| 79.91 (15.01) \| 84.03 (17.57) \| 78.42 (14.44) \|  \| 78.62 (11.56) \| 80.35 (10.69) \| 77.73 (11.37) \| \| Valence \|  \| 7.12 (.99) \| 6.33 (1.38) \| 6.85 (1.20) \|  \| 6.81 (1.20) \| 6.27 (1.25) \| 6.31 (1.41) \| \| Arousal \|  \| 3.61 (1.60) \| 5.27 (2.02) \| 3.42 (1.52) \|  \| 2.73 (1.56) \| 3.96 (1.51) \| 3.23 (1.90) \| \| Expected plausibility* \|  \| - \| 4.58 (1.20) \| 4.42 (1.39) \|  \| - \| 4.50 (0.86) \| 4.04 (1.31) \| \| Perceived plausibility \|  \| - \| 4.39 (0.97) \| 4.03 (1.16) \|  \| - \| 4.08 (0.89) \| 3.88 (1.14) \|   *Footnote:* SBP: Systolic blood pressure; DBP: Diastolic blood pressure; HR: Heart rate; * of arguments |
| --- | --- | --- | --- | --- | --- | --- | --- | --- | --- | --- | --- | --- | --- | --- | --- | --- | --- | --- | --- | --- | --- | --- | --- | --- | --- | --- | --- | --- | --- | --- | --- | --- | --- | --- | --- | --- | --- | --- | --- | --- | --- | --- | --- | --- | --- | --- | --- | --- | --- | --- | --- | --- | --- | --- | --- | --- | --- | --- | --- | --- | --- | --- | --- | --- | --- | --- | --- | --- | --- | --- | --- | --- | --- | --- | --- | --- | --- | --- | --- | --- | --- | --- | --- | --- | --- | --- | --- | --- | --- | --- |

**Table S2** Effects found in the analyses of covariance

|  |  | | Effect | | | | |
| --- | --- | --- | --- | --- | --- | --- | --- |
| Measure |  | Group | | Time | Group x time | Covariate | Time x covariate |
| SBP |  | F(1,56) = 5.71, p = .020*,  η2p = .092 | | F(1,56) = 0.30,  p = .585,  η2p = .005 | F(1,56) = 0.09,  p = .767,  η2p = .002 | F(1,56) = 57.99, p<.001,  η2p = .509 | F(1,56) = 0.59,  p = .444,  η2p = .010 |
| DBP |  | F(1,56) = 2.35, p =.131,  η2p = .040 | | F(1,56) = 0.14,  p = .714,  η2p = .002 | F(1,56) =0.13,  p = .720,  η2p = .002 | F(1,56) = 39.10,  p < .001,  η2p = .411 | F(1,56) = 0.19,  p = .662,  η2p = .003 |
| HR |  | F(1,56) = 0.33, p= .566,  η2p = .006 | | F(1,56) = 0.11,  p = .738,  η2p = .002 | F(1,56) = 1.92, p = .172,  η2p = .033 | F(1,56) = 141.25, p < .001,  η2p = .716 | F(1,56) = 1.02., p =.316,  η2p =.018 |
| Arousal |  | F(1,56) = 0.37,  p = .545,  η2p =.007 | | F(1,56) = 0.07,  p = 789.,  η2p =.001 | F(1,56) = 3.00, p = .089,  η2p = .051 | F(1,56) = 46.37,  p < .001,  η2p = .453 | F(1,56) = 6.64, p = .013*,  η2p = .106 |
| Valence |  | F(1,56) = 0.06, p = .806,  η2p = .001 | | F(1,56) = 0.65,  p = .423,  η2p = .012 | F(1,56) = 2.81, p = .099,  η2p = .048 | F(1,56) = 62.92,  p < .001,  η2p =.529 | F(1,56) = 0.28,  p = .601,  η2p = .005 |

*Footnote*. SBP: Systolic blood pressure; DBP: Diastolic blood pressure; HR: Heart rate; * denotes significance at the .05 level

**Table S3** Effects found in the 2x2 mixed analyses of variance

|  |  | Effect | | |
| --- | --- | --- | --- | --- |
| Measure |  | Group | Time | Group x time |
| Confidence in plausibility of arguments |  | F(1,57) = 0.67,  p = .417,  η2p = .012 | F(1,57) = 4.17, p = .046*,  η2p = .068 | F(1,57) = 1.07,  p =.306,  η2p = .018 |
| Confidence that arguments are seen as plausible by others |  | F(1,57) = 0.88,  p = .351,  η2p = .015 | F(1,57) = .5.16, p = .027*,  η2p = .083 | F(1,57) =0.49,  p = .487,  η2p =.009 |

*Footnote*. * denotes significance at the .05 level

**Supplementary Material: Scales with English translations**

**Social Interaction Anxiety Scale (SIAS) German Version**

Bitte geben Sie in den folgenden Aussagen an, in welchem Ausmaß diese auf Sie zutreffen. Kreuzen Sie möglichst bei jeder Frage eine Zahl an. Dabei bedeutet:

0 = überhaupt nicht zutreffend
1 = ein wenig zutreffend
2 = ziemlich zutreffend
3 = stark zutreffend
4 = sehr stark zutreffend


*English translation of instruction:*
*In the following statements, please indicate the extent to which these apply to you. If possible, mark a number with a cross for each question. Whereby means:*

*0 = not applicable at all*

*1 = somewhat applicable*

*2 = fairly applicable*

*3 = strongly applicable*

*4 = very strongly applicable*

| 1. Ich werde nervös, wenn ich mit einer Autoritätsperson (Lehrer, Vorgesetzten) sprechen muss. | 0 | 1 | 2 | 3 | 4 |
| --- | --- | --- | --- | --- | --- |
| Engl.: I get nervous if I have to speak to someone in authority (teacher, superiors). |  |  |  |  |  |
| 1. Ich habe Schwierigkeiten, Blickkontakt mit anderen herzustellen. |  |  |  |  |  |
| Engl.: I have difficulty having eye contact with others. |  |  |  |  |  |
| 1. Ich werde angespannt, wenn ich über mich oder meine Gefühle sprechen muss. |  |  |  |  |  |
| Engl.: I become tense if I have to talk about myself or my feelings. |  |  |  |  |  |
| 1. Ich finde es schwierig, mich im Umgang mit Leuten, mit denen ich zusammenarbeite, wohlzufühlen. |  |  |  |  |  |
| Engl.: I find it difficult to feel comfortable interacting with people I work with. |  |  |  |  |  |
| 1. Mir fällt es leicht, Freunde in meinem Alter zu finden. |  |  |  |  |  |
| Engl.: I find it easy to make friends my own age. |  |  |  |  |  |
| 1. Ich werde angespannt, wenn ich einen Bekannten auf der Straße treffe. |  |  |  |  |  |
| Engl.: I tense up if I meet an acquaintance in the street. |  |  |  |  |  |
| 1. Wenn ich mit anderen zusammen bin, fühle ich mich unwohl. |  |  |  |  |  |
| Engl.: When I am with others, I feel uncomfortable. |  |  |  |  |  |
| 1. Ich fühle mich angespannt, wenn ich mit einer Person alleine zusammen bin. |  |  |  |  |  |
| Engl.: I feel tense if I am alone with just one other person. |  |  |  |  |  |
| 1. Ich fühle mich wohl, wenn ich Leute bei Parties usw. kennenlerne. |  |  |  |  |  |
| Engl.: I am at ease meeting people at parties, etc. |  |  |  |  |  |
| 1. Es fällt mir schwer, mich mit anderen Leuten zu unterhalten. |  |  |  |  |  |
| Engl.: I have difficulty talking with other people. |  |  |  |  |  |
| 1. Mir fallen leicht Dinge ein, über die man reden kann. |  |  |  |  |  |
| Engl.: I find it easy to think of things to talk about. |  |  |  |  |  |
| 1. Ich mache mir Sorgen, dass ich durch meine Äußerungen ungeschickt erscheinen könnte. |  |  |  |  |  |
| Engl.: I'm worried that my remarks might make me seem awkward. |  |  |  |  |  |
| 1. Mir fällt es schwer, einen anderen Standpunkt als andere zu vertreten. |  |  |  |  |  |
| Engl.: I find it difficult to disagree with another’s point of view. |  |  |  |  |  |
| 1. Ich finde es schwierig, mit einer attraktiven Person des anderen Geschlechts zu reden. |  |  |  |  |  |
| Engl.: I have difficulty talking to an attractive person of the opposite sex. |  |  |  |  |  |
| 1. Es kommt vor, dass ich mir darüber Sorgen mache, in sozialen Situationen nicht zu wissen, was ich sagen könnte. |  |  |  |  |  |
| Engl.: I find myself worrying that I won’t know what to say in social situations. |  |  |  |  |  |
| 1. Ich bin nervös, wenn ich Leute treffe, die ich nicht gut kenne. |  |  |  |  |  |
| Engl.: I'm nervous when I meet people I don't know well. |  |  |  |  |  |
| 1. Ich glaube immer, dass ich beim Reden etwas Peinliches sagen könnte. |  |  |  |  |  |
| Engl.: I always feel I’ll say something embarrassing while talking. |  |  |  |  |  |
| 1. Wenn ich zu einer Gruppe dazukomme, mache ich mir Sorgen, dass ich ignoriert werden könnte. |  |  |  |  |  |
| Engl.: When I join a group, I worry about being ignored. |  |  |  |  |  |
| 1. Ich fühle mich angespannt, wenn ich zu einer Gruppe dazukomme. |  |  |  |  |  |
| Engl.: I feel tense when I join a group. |  |  |  |  |  |
| 1. Ich bin unsicher, ob ich jemanden grüßen soll, den ich nur oberflächlich kenne. |  |  |  |  |  |
| Engl.: I am unsure whether to greet someone I know only superficially. . |  |  |  |  |  |

**Furcht vor negativer Evaluation – Kurzskala (FNE-K)**

Bitte lesen Sie jede der folgenden Feststellungen aufmerksam durch und geben Sie durch Ankreuzen auf der angegebenen Skala an, wie charakteristisch diese ihrer Meinung nach für Sie ist.

*1 = überhaupt nicht charakteristisch für mich, 2 = ein bisschen charakteristisch für mich, 3 = einigermaßen charakteristisch für mich, 4 = sehr charakteristisch für mich, 5. äußerst charakteristisch für mich*

*English translation of instruction:*
Please read each of the following statements carefully and indicate how characteristic you think it is for you by marking it with a cross on the scale provided.

1 = not characteristic of me at all, 2 = somewhat characteristic of me, 3 = somewhat characteristic of me, 4 = very characteristic of me, 5. extremely characteristic of me

| 1. Ich mache mir Gedanken darüber, was andere Leute von mir denken, auch wenn ich weiß, dass es egal ist. | 1 | 2 | 3 | 4 | 5 |
| --- | --- | --- | --- | --- | --- |
| Engl.: I worry about what other people will think of me even when I know it doesn’t make any difference. |  |  |  |  |  |
| 1. Es bekümmert mich, wenn ich merke, dass andere Leute einen schlechten Eindruck von mir bekommen. |  |  |  |  |  |
| Engl.: It bothers me when people form an unfavorable impression of me. |  |  |  |  |  |
| 1. Ich habe oft Angst, dass andere Leute meine Fehler bemerken. |  |  |  |  |  |
| Engl.: I am frequently afraid of other people noticing my mistakes. |  |  |  |  |  |
| 1. Ich mache mir öfters Gedanken darüber, welchen Eindruck ich auf jemand anderes mache. |  |  |  |  |  |
| Engl.: I worry about what kind of impression I make on people. |  |  |  |  |  |
| 1. Ich habe Angst, dass andere sich nicht positiv über mich äußern. |  |  |  |  |  |
| Engl.: I am afraid that others will not speak positively about me. |  |  |  |  |  |
| 1. Ich habe Angst, dass andere Leute etwas an mir auszusetzen haben. |  |  |  |  |  |
| Engl.: I am afraid that other people will find fault with me. |  |  |  |  |  |
| 1. Die Meinung anderer Leute über mich ist mir wichtig. |  |  |  |  |  |
| Engl.: Other people's opinion about me is important to me. |  |  |  |  |  |
| 1. Wenn ich mit jemandem spreche, mache ich mir Gedanken darüber, was der andere über mich denken könnte. |  |  |  |  |  |
| Engl.: When I am talking to someone, I worry about what they may be thinking about me. |  |  |  |  |  |
| 1. Normalerweise mache ich mir Gedanken darüber, wie ich auf andere wirke. |  |  |  |  |  |
| Engl.: I am usually worried about what kind of impression I make. |  |  |  |  |  |
| 1. Es macht mir etwas aus, wenn ich weiß, dass mich jemand beurteilt. |  |  |  |  |  |
| Engl.: It bothers me when I know someone is judging me. |  |  |  |  |  |
| 1. Manchmal glaube ich, ich beschäftige mich viel zu sehr damit, was andere Leute von mir denken. |  |  |  |  |  |
| Engl.: Sometimes I think I am too concerned with what other people think of me. |  |  |  |  |  |
| 1. Ich habe oft Angst, dass ich etwas Falsches sagen oder tun würde. |  |  |  |  |  |
| Engl.: I am often afraid that I will say or do something wrong. |  |  |  |  |  |

**Brief Symptom Inventory-18 (BSI-18) German Version**

Sie finden nachstehend eine Liste von Problemen und Beschwerden, die man manchmal hat. Bitte lesen Sie jede Frage einzeln sorgfältig durch und entscheiden Sie, wie stark Sie durch diese Beschwerden gestört oder bedrängt worden sind, und zwar während der vergangenen sieben Tage bis heute. Überlegen Sie bitte nicht erst, welche Antwort „den besten Eindruck“ machen könnte, sondern antworten Sie so, wie es für Sie persönlich zutrifft. Machen Sie bitte hinter jeder Frage ein Kreuz bei der für Sie am besten zutreffenden Antwort.


Wie sehr litten Sie in den letzten sieben Tagen unter:


*English translation of instruction:*
You will find below a list of problems and complaints that you sometimes have. Please read each question carefully one by one and decide how much you have been disturbed or distressed by these complaints, during the past seven days until today. Please do not think first about which answer might make the best impression, but answer as it applies to you personally. After each question, please put a cross by the answer that best applies to you.

How much did you suffer in the last seven days from

0 = not at all

1 = a little

2 = quite

3 = strongly

4 = very strongly

0 = überhaupt nicht

1 = ein wenig

2 = ziemlich

3 = stark

4 = sehr stark

| 1. Ohnmachts- und Schwindelgefühl | 0 | 1 | 2 | 3 | 4 |
| --- | --- | --- | --- | --- | --- |
| Engl.: Feeling faint and dizzy. |  |  |  |  |  |
| 1. dem Gefühl, sich für nichts zu interessieren |  |  |  |  |  |
| Engl.: The feeling of not being interested in anything. |  |  |  |  |  |
| 1. Nervosität oder innerem Zittern |  |  |  |  |  |
| Engl.: Nervousness or shakiness inside |  |  |  |  |  |
| 1. Herz- oder Brustschmerzen |  |  |  |  |  |
| Engl.: Pains in heart or chest |  |  |  |  |  |
| 1. Einsamkeitsgefühlen |  |  |  |  |  |
| Engl.: Feelings of loneliness |  |  |  |  |  |
| 1. dem Gefühl, gespannt oder aufgeregt zu sein |  |  |  |  |  |
| Engl.: Feeling tensed or keyed up |  |  |  |  |  |
| 1. Übelkeit/ Magenverstimmung |  |  |  |  |  |
| Engl.: Nausea or upset stomach |  |  |  |  |  |
| 1. Schwermut |  |  |  |  |  |
| Engl.: Melancholy |  |  |  |  |  |
| 1. Erschrecken ohne Grund |  |  |  |  |  |
| Engl.: Suddenly scared for no reason |  |  |  |  |  |
| 1. Schwierigkeiten beim Atmen |  |  |  |  |  |
| Engl.: Breathing difficulties |  |  |  |  |  |
| 1. dem Gefühl, wertlos zu sein |  |  |  |  |  |
| Engl.: Feeling of worthlessness |  |  |  |  |  |
| 1. Schreck- oder Panikanfällen |  |  |  |  |  |
| Engl.: Fright or panic attacks |  |  |  |  |  |
| 1. Taubheit oder Kribbeln in einzelnen Körperteilen |  |  |  |  |  |
| Engl.: Numbness or tingling in individual parts of the body |  |  |  |  |  |
| 1. einem Gefühl der Hoffnungslosigkeit angesichts der Zukunft |  |  |  |  |  |
| Engl.: Feeling hopeless about the future |  |  |  |  |  |
| 1. Ruhelosigkeit |  |  |  |  |  |
| Engl.: Restlessness |  |  |  |  |  |
| 1. Schwächegefühl in einzelnen Körperteilen |  |  |  |  |  |
| Engl.: Feeling of weakness in individual parts of the body |  |  |  |  |  |
| 1. Gedanken, sich das Leben zu nehmen |  |  |  |  |  |
| Engl.: Thoughts to take your own life |  |  |  |  |  |
| 1. Furchtsamkeit |  |  |  |  |  |
| Engl.: Fearfulness |  |  |  |  |  |
